# Aspirin in Primary Prevention of CVD: A Pragmatic Outline and Advice for Treatment Personalization

**DOI:** 10.3390/jcm15155936

**Published:** 2026-07-29

**Authors:** Francesca Santilli, Dirk Sibbing, Augusto Maria Lavalle Cobo

**Affiliations:** 1 Department of Medicine and Aging and Center for Advanced Studies and Technology, University of Chieti-Pescara, Via Luigi Polacchi 11, 66100 Chieti, Italy; 2 Department of Cardiology, Ludwig-Maximilians-Universität (LMU) München, Geschwister-Scholl-Platz 1, 80539 Münich, Germany; 3 Privatklinik Lauterbacher Mühle am Ostersee, Unterlauterbach 1, 82402 Seeshaupt, Germany; 4 3DZHK (German Center for Cardiovascular Research), Partner Site Münich Heart Alliance, Lazarettstraße 36, 80636 Münich, Germany; 5 Cardiology Department, Sanatorio Otamendi, Ciudad Autonoma de Buenos Aires C1007ABJ, Argentina

**Keywords:** low-dose aspirin, cardiovascular disease, primary prevention, advanced subclinical atherosclerosis

## Abstract

Cardiovascular disease (CVD) remains the leading global cause of morbidity and mortality. While low-dose aspirin is well established for secondary prevention, its role in primary prevention is controversial because modest benefits in cardiovascular benefits must be balanced against increased bleeding risk, as highlighted by recent large trials. Current guidelines recommend restricting aspirin use to individuals at higher cardiovascular risk without elevated bleeding risk, yet significant heterogeneity persists in defining these thresholds. Evidence from randomized trials, guidelines, cohort and modeling analyses, and post hoc or observational subgroup studies suggests that selected patients with advanced subclinical atherosclerosis or specific high-risk markers may have a more favorable risk–benefit profile, although the certainty and directness of evidence vary across subgroups. This narrative review and pragmatic clinical guide presents an illustrative tiered framework based on clinical, imaging, and biomarker markers—such as coronary artery calcium score, carotid plaque score, and lipoprotein(a)—to support individualized discussion of preventive aspirin therapy. The framework is not a validated risk calculator or standalone treatment algorithm, and decisions should balance absolute cardiovascular risk, major bleeding risk, evidence certainty, and patient preferences.

## 1. Introduction

Cardiovascular disease (CVD) remains the leading cause of morbidity and mortality worldwide, accounting for almost one-third of all global deaths in 2022 [1]. The management of CVD risk is traditionally divided into primary prevention for individuals without prior events and secondary prevention for those with established CVD. There is a growing recognition of an intermediate group with advanced subclinical atherosclerosis who, despite no prior clinical events, faces high risk of initial or recurrent incidents [2].

Aspirin is the established antiplatelet agent for secondary CVD prevention due to its net clinical benefit, but its use in primary prevention is currently debated. In 2018, three large randomized controlled trials—ASCEND [3], ASPREE [4], and ARRIVE [5]—challenged the paradigm by demonstrating only modest benefits alongside increased bleeding risk. These findings prompted a reassessment of aspirin’s risk–benefit profile in lower-risk groups. As a result, major guidelines now advise limiting aspirin for primary prevention to individuals with higher cardiovascular risk and no elevated bleeding risk [6,7]. Yet, considerable heterogeneity persists across these international guidelines, both in the risk thresholds applied and in the interpretation of what constitutes “elevated risk,” creating uncertainty for clinicians attempting to identify appropriate candidates for primary prevention aspirin therapy [8].

Emerging evidence suggests that patients at a higher risk of a primary event, particularly those with advanced subclinical atherosclerosis, may represent a unique high-risk group for whom aspirin could confer net clinical benefit [2]. Notably, many of these trials did not apply risk-score-based inclusion criteria that would have enabled clear identification of patients at high or very high cardiovascular risk.

These individuals often remain classified within the primary prevention category by conventional criteria but share risk characteristics (and arguably prognosis) closer to those in the secondary prevention category. For such patients, the absolute reduction in cardiovascular events when using aspirin may outweigh bleeding risk, underscoring the importance of nuanced risk stratification and individualized decision-making.

This article seeks to provide practical advice for primary care physicians on identifying subgroups of patients who may derive net benefit from preventive aspirin therapy. Using existing evidence, we propose a tiered benefit framework that integrates emerging signals across traditionally siloed domains. While risk score calculators can guide the decision, as previously mentioned, the major primary prevention trials did not use these. In this framework, patients are categorized as having moderate, mild, or low/no net benefit, based on clinical factors and advanced imaging markers. This approach aligns with the broader movement toward individualized prevention strategies and may bridge the gap between population-based guideline recommendations and patient-centered cardiovascular risk management.

## 2. Methods

This article is intended as a narrative review and pragmatic clinical guide rather than a systematic review. Relevant evidence was identified from major randomized controlled trials, guideline documents, meta-analyses, cohort studies, and post hoc analyses addressing aspirin use for primary prevention of CVD. Evidence from randomized trials and contemporary guidelines was prioritized when available, whereas observational and post hoc subgroup analyses were interpreted as hypothesis-generating. The proposed framework was developed to synthesize these heterogeneous evidence sources into a practical decision aid for primary care physicians and should not be interpreted as a validated risk calculator. Because the supporting evidence varies by subgroup, recommendations are presented according to the directness and certainty of the evidence rather than as uniform treatment recommendations.

## 3. Conceptual Framework for Determining the Net Clinical Benefit of Aspirin

The proposed framework (Table 1) is intended as an illustrative clinical decision aid to support individualized assessment of potential net benefit. The categories reflect the relative strength and directness of available evidence, integrating cardiovascular risk, bleeding risk, and the nature of supporting data. The proposed thresholds were selected according to the directness of supporting evidence. CAC ≥ 100 reflects cohort analyses, risk modeling, and guideline-supported risk stratification suggesting higher ASCVD risk and potential aspirin benefit when bleeding risk is low. CPS ≥ 2 is based on MESA and ARIC analyses and has not been tested in dedicated randomized aspirin trials. Elevated Lp(a), defined as ≥50 mg/dL or ≥125 nmol/L, is supported by post hoc and observational analyses suggesting possible aspirin-associated risk reduction. These thresholds are therefore pragmatic risk-stratification markers rather than validated treatment cut-offs. Net clinical benefit was conceptualized as the expected absolute reduction in ASCVD events weighed against the expected absolute increase in major bleeding, considering absolute risk reduction, bleeding rates, number needed to treat, and number needed to harm where available, but not as a formal scoring system.

## 4. Assessing Cardiovascular Risk

### Age and CVD Risk

Table 2 summarizes the factors to be considered when assessing CVD risk to identify patients who may benefit from aspirin. Along with other modifiable and non-modifiable factors, age remains one of the strongest risk factors for CVD [9]. Overall, the net clinical benefit of aspirin is greater at a younger age [4], and the ASPREE trial and its recent follow-up [10] have led to downgrading of aspirin use for primary prevention in older adults. This is reflected in the 2019 guidelines of the American College of Cardiology (ACC) and American Heart Association (AHA), which recommend that aspirin should not be used on a routine basis for primary prevention of atherosclerotic cardiovascular disease (ASCVD) in adults aged >70 years [6]. However, some authors have criticized aspects of ASPREE’s design and analysis, such as population selection and event rates, which leaves room for nuanced, individualized consideration [11].

Although the US Preventive Services Task Force recommends against aspirin use for primary prevention in adults aged 60 years or older, a personalized approach may still justify aspirin therapy for selected patients at very high cardiovascular risk based on clinical factors or subclinical atherosclerosis. In such cases, general practitioners should consider the overall risk profile and make patient-centered decisions. This should include issues relating to aspirin discontinuation, which is often associated with increased risk of major adverse cardiovascular events (MACE) possibly due to forgone benefit or rebound effect [10,12].

## 5. Advanced Subclinical Atherosclerosis Risk Factors or Soluble Biomarkers

### 5.1. Coronary Artery Calcium Score

Coronary artery calcium (CAC) is a highly specific feature of coronary atherosclerosis, making CAC scoring a reliable and widely used method for assessing cardiovascular risk, especially in asymptomatic individuals. For example, the ACC/AHA guidelines recommend CAC scoring to assist doctor–patient risk discussions in patients at intermediate or borderline risk [6]. CAC scoring supports primary prevention strategies, such as statins and aspirin, and is cost effective across different baseline risks according to studies with up to 15-year follow-up data [13].

The evidence supporting CAC-guided aspirin allocation is derived primarily from cohort analyses, risk modeling, and guideline-supported risk stratification rather than randomized trials specifically designed to test CAC-guided aspirin initiation.

Individuals with CAC scores of ≥100 (especially ≥ 400) are most likely to experience a net benefit from aspirin therapy; high CAC scores correlate with significantly higher ASCVD risk, and aspirin can reduce cardiovascular events in patients with low bleeding risk.

Clinical benefit is uncertain in individuals with intermediate CAC scores (1–99), so further stratification based on clinical factors including age, carotid plaque score (CPS), and concomitant conditions (e.g., type 2 diabetes mellitus [T2DM] and chronic kidney disease [CKD]) is required.

Aspirin use in individuals with a CAC score of zero is generally discouraged, as bleeding risk outweighs any cardiovascular benefit in this group [13,14,15].

### 5.2. Carotid Plaque Score

CPS is derived from ultrasound imaging of the carotid arteries and is emerging as a practical and evidence-based tool to guide aspirin use in primary prevention of CVD. An analysis of patients from the MESA study found that a CPS of ≥2 was associated with a net clinical benefit from aspirin therapy, balancing reduced CVD events against bleeding risk [16].

This evidence is based on analyses from MESA and ARIC rather than dedicated randomized aspirin trials, and CPS should therefore be viewed as an emerging risk-stratification marker requiring further validation before routine use as a standalone aspirin-allocation tool.

### 5.3. Lipoprotein(a)

Lipoprotein(a) (Lp[a]) is a promising biomarker to guide aspirin therapy for primary prevention of CVD. In the MESA study, individuals with Lp(a) ≥ 50 mg/dL demonstrated a 46% lower risk of coronary heart disease with aspirin use, while no benefit was observed in those with lower Lp(a) levels [17]. In the NHANES III study, aspirin was associated with a 52% reduction in ASCVD mortality, with a significant interaction between Lp(a) and aspirin use observed [18].

These data should be interpreted cautiously because they derive from post hoc and observational subgroup analyses rather than prospective randomized trials designed to evaluate whether elevated Lp(a) modifies the effect of aspirin.

Patients with elevated Lp(a) levels (≥50 mg/dL or 125 nmol/L) may have an increased risk of MACE and may be considered for individualized discussion of aspirin therapy, particularly when other cardiovascular risk markers are present and bleeding risk is low. These findings should be interpreted cautiously because the available evidence derives from post hoc and observational subgroup analyses rather than randomized trials specifically designed to test an aspirin–Lp(a) interaction. While biologically plausible, this interaction requires prospective validation in a dedicated randomized trial before elevated Lp(a) can be recommended as a standalone decision-making tool for aspirin initiation.

### 5.4. Coronary Plaque Burden

The presence of coronary plaque burden, identified by coronary computed tomography, may signal a similar profile to the secondary prevention category [19].

### 5.5. Other Risk Biomarkers

Use of traditional CVD risk factors alone may have limitations in the extent of discrimination between risk categories. More recently, cardiovascular biomarkers such as cardiac troponins, natriuretic peptides, and inflammatory markers may be used to enhance CVD risk classification. However, routine use in primary practice is currently limited, and further clinical evidence is required to demonstrate their impact on reducing cardiovascular events [20].

## 6. Other Signals of Interest

### 6.1. Type 2 Diabetes Mellitus

Patients with diabetes have an elevated risk of atherosclerotic cardiovascular events, and silent coronary artery disease is common in people with T2DM [21,22]. Identifying patients with diabetes who have subclinical ischemia and higher cardiovascular risk is crucial, as their risk may warrant more aggressive preventive measures such as aspirin [23].

Current treatment guidelines suggest a nuanced approach to aspirin use in patients with diabetes. The American Diabetes Association recommends low-dose aspirin in adults aged 50–70 years who are not at an increased risk of bleeding and have at least one major CVD risk factor [24]. Similarly, joint guidelines from the European Society of Cardiology and European Association for the Study of Diabetes recommend low-dose aspirin in those at very high/high risk in the absence of clear contraindications [25].

While aspirin can reduce serious vascular events in patients with diabetes and no CVD, an increase in major bleeding negates the overall benefit [3].

Recent evidence questions the benefit of low-dose aspirin to prevent CVD in patients with T2DM. While aspirin can reduce serious vascular events in patients with diabetes and no CVD, an increase in major bleeding negates the overall benefit [3]. Over a 7.4-year follow-up, ASCEND reported a 12% proportional reduction in serious vascular events, which was nearly offset by a 0.9% absolute increase in major bleeding. This long-term outcome contrasts with the first three years of treatment, where the risk reduction is typically much higher, at approximately 25%, mirroring what is observed in secondary prevention. This apparent attenuation over time may simply reflect the progressive reduction in the size of the population remaining at risk after the initial years. Proton pump inhibitor (PPI) co-therapy may improve the safety profile of aspirin by reducing upper gastrointestinal bleeding risk in selected patients with elevated gastrointestinal bleeding risk, but it does not mitigate other serious bleeding outcomes. Overall, aspirin may be considered for adults with diabetes who are at increased CVD risk. However, a discussion with the patient on the risk of bleeding and the potential for only limited benefit of treatment is essential.

### 6.2. Chronic Kidney Disease

Several meta-analyses have reported that aspirin use in patients with CKD is associated with cardiovascular benefits and overall net benefit, particularly for patients in the later stages of the disease [26,27]. Oral low-dose aspirin is therefore recommended for secondary prevention of recurrent ischemic cardiovascular events in people with CKD and established ischemic CVD (Grade 1C) [28]. In cases of aspirin intolerance, other antiplatelet therapies such as P2Y12 inhibitors should be considered.

For CKD, the evidence base and recommendations should be distinguished by prevention setting: support for aspirin is strongest for secondary prevention in patients with established ischemic CVD, whereas primary prevention evidence in CKD remains less direct and must be balanced carefully against the higher bleeding risk in this population.

### 6.3. Metabolic Dysfunction-Associated Steatotic Liver Disease

In patients with metabolic dysfunction-associated steatotic liver disease (MASLD) without cirrhosis, low-dose aspirin has been shown to significantly reduce liver fat compared with placebo [29]. However, the short study duration and exclusion of advanced liver disease limit the generalizability of these findings [30]. Aspirin use may also be associated with chemoprevention of hepatocellular carcinoma in patients with MASLD [31]. Overall, however, the use of aspirin for patients with chronic liver disease or pancreatitis should be avoided until further evidence is available, unless there is a compelling indication.

The randomized MASLD evidence relates primarily to short-term changes in liver fat rather than cardiovascular event prevention, so it should not be interpreted as direct evidence supporting aspirin for primary prevention of CVD in MASLD.

### 6.4. Familial Hypercholesterolemia

Low-density lipoprotein cholesterol concentrations are markedly increased from birth in patients with familial hypercholesterolemia, which, if left untreated, can lead to ASCVD. In the presence of ASCVD risk factors (cardiovascular risk, elevated Lp[a], or diabetes), low-dose aspirin may be considered for primary prevention [32].

## 7. Balancing Bleeding Risk

While low-dose aspirin may benefit select individuals with advanced subclinical atherosclerosis, its use must always be weighed against the potential for bleeding complications. Aspirin irreversibly inhibits platelet cyclooxygenase-1, reducing thromboxane A_2_-mediated platelet aggregation, but simultaneously impairs physiological hemostasis [33]. This mechanism underpins aspirin’s cardiovascular benefit but also contributes to an increased risk of gastrointestinal bleeding, intracranial hemorrhage, and hemorrhagic stroke [34].

Population-based studies have identified several predictors of aspirin-associated bleeding, including advanced age (≥75 years), Asian or Pacific ancestry compared with European ancestry, current smoking, and a history of diabetes, cancer, prior bleeding, peptic ulcer disease, alcohol-related disorders, chronic liver disease, or pancreatitis [34]. Concomitant use of nonsteroidal anti-inflammatory drugs, corticosteroids, selective serotonin reuptake inhibitors, or medications for peptic ulcer disease further amplifies this risk [34]. Recognizing and quantifying these factors are essential before initiating long-term aspirin therapy (Table 3).

Unlike the management of patients with atrial fibrillation (AF) or acute coronary syndrome (ACS), where bleeding risk calculators have been developed and validated, these tools have not been validated for use in patients receiving aspirin in the primary prevention setting. In this context, reliable risk-scoring calculators are currently unavailable; therefore, it is suggested to identify individual variables associated with a higher risk of bleeding, many of which are already included in established scores such as HAS-BLED and CRUSADE.

Several strategies may mitigate upper gastrointestinal bleeding risk. PPIs significantly reduce upper gastrointestinal bleeding incidence [35] and may therefore be considered in patients who require aspirin but possess elevated gastrointestinal bleeding risk, particularly older adults or those with a prior history of ulcer disease. Additionally, *Helicobacter pylori* eradication has demonstrated short-term benefit in preventing peptic ulcer bleeding among aspirin users, though long-term protective effects remain uncertain [36]. In individuals with high CVD risk and documented *H. pylori* infection, eradication therapy may represent a reasonable adjunctive measure. However, PPI therapy and *H. pylori* eradication should not be assumed to mitigate other serious bleeding outcomes, including intracranial hemorrhage.

A personalized approach that addresses modifiable risk factors and includes prophylactic measures allows for safer aspirin therapy, maximizing cardiovascular benefits while minimizing bleeding risks.

## 8. Practical Steps for Decision-Making

Aspirin’s low cost, once-daily regimen may be relevant in settings where comprehensive risk-factor modification is challenging; however, aspirin should not be viewed as a substitute for lifestyle intervention, blood pressure control, lipid-lowering therapy, smoking cessation, or diabetes management. Any potential increase in absolute cardiovascular benefit in higher-risk populations must be weighed against the possibility of similarly elevated background bleeding risk, including undiagnosed anemia, peptic ulcer disease, or *H. pylori* infection. From a pharmacologic perspective, low-dose aspirin (75–100 mg daily) is sufficient to achieve effective platelet inhibition; higher doses have not demonstrated incremental cardiovascular protection but are associated with increased bleeding risk [37]. When considering aspirin initiation, clinicians should first estimate the patient’s ASCVD risk, using traditional clinical factors and advanced subclinical markers such as CAC scoring, alongside individual bleeding risk. The proposed framework (Table 1) provides a practical way to balance these competing risks. Physicians should clearly discuss the personalized benefits and risks of aspirin with patients, considering their cardiovascular and bleeding risks. Visual tools showing event probabilities may improve understanding and adherence. Finally, patient suitability for aspirin should be periodically reviewed, considering any bleeding manifestation as well as advanced atherosclerosis risk factors and/or modifiers, as these parameters evolve with age, comorbidities, and concomitant therapies.

## 9. Conclusions

Aspirin’s role in primary CVD prevention is still debated, with evolving evidence making traditional “primary” and “secondary” categories less useful. Research suggests that patients with advanced subclinical atherosclerosis may benefit from low-dose aspirin when risk is carefully assessed and bleeding risks are minimized. Importantly, the extent to which other modifiable risk factors—such as obesity, smoking, diabetes, and dyslipidemia—are successfully addressed is often an underestimated determinant in the decision to initiate aspirin therapy. In settings where patients are noncompliant or where socioeconomic barriers limit access to weight loss interventions, smoking cessation resources, or optimal management of diabetes and cholesterol, the relative contribution of aspirin to absolute risk reduction may be larger; however, this remains a hypothesis that requires further study and must be balanced against local bleeding-risk profiles. Aspirin should therefore be considered only as part of a broader prevention strategy that prioritizes lifestyle intervention, lipid and blood pressure control, smoking cessation, and diabetes management. By adopting a structured, evidence-based framework and embracing shared decision-making, primary care physicians can more confidently interpret their local guidelines and navigate this nuanced therapeutic territory, ensuring that preventive aspirin use is both targeted and rational to maximize cardiovascular protection while minimizing potential harm.

## Figures and Tables

**Table 1:** Illustrative framework for determining the net clinical benefit of aspirin.

Moderate Net Benefit ++	Mild * Net Benefit +	No Net Benefit −
**Advanced subclinical atherosclerosis** Defined as: CAC score ≥ 100, or CPS ≥ 2	**≥1 comorbidity:** T2DM with low bleeding risk Stage 3 to 5 CKD MASLD FH	**≥1 characteristic:** CAC score = 0 No carotid plaque High bleeding risk ^†^
	**OR** **Elevated Lp(a)** **≥125 nmol/L**	

The categories are not derived from a single validated scoring system. They reflect synthesis of randomized trial evidence, guideline recommendations, cohort and modeling analyses, observational studies, post hoc subgroup analyses, and expert interpretation. Net clinical benefit was conceptualized as the expected absolute reduction in ASCVD events weighed against the expected absolute increase in major bleeding, with consideration of absolute risk reduction, bleeding rates, number needed to treat, and number needed to harm where available. * If several features of mild benefit coexist, the patient may shift toward the moderate benefit category. ^†^ In patients at higher bleeding risk, *Helicobacter pylori* should be eradicated and PPI therapy considered. These measures may reduce upper gastrointestinal bleeding risk but should not be assumed to mitigate other serious bleeding outcomes, including intracranial hemorrhage. CAC, coronary artery calcium; CKD, chronic kidney disease; CPS, carotid plaque score; FH, familial hypercholesterolemia; Lp(a), lipoprotein(a); MASLD, metabolic dysfunction-associated steatotic liver disease; PPI, proton pump inhibitor.

**Table 2:** Factors to consider when assessing CVD risk.

Factor/Risk Marker	Description/Clinical Relevance	Example/Notes
Age	One of the strongest risk factors for CVD; risk increases with age	Net benefit of aspirin is generally greater at a younger age
Coronary artery calcium (CAC) score	Highly specific marker of coronary atherosclerosis; supports risk stratification for primary prevention	CAC ≥ 100 (especially ≥400) indicates higher risk and potential net benefit from aspirin
Carotid plaque score (CPS)	Ultrasound-based score; emerging tool for guiding aspirin use in primary prevention	CPS ≥ 2 has been associated with estimated net clinical benefit in MESA/ARIC analyses; validation in dedicated aspirin trials is lacking
Lipoprotein(a) (Lp[a])	Elevated levels (≥50 mg/dL or 125 nmol/L) linked to increased risk of major adverse cardiovascular events	Aspirin benefit has been suggested in post hoc/observational analyses of individuals with elevated Lp(a)
Coronary plaque burden	Identified by coronary CT; may indicate risk profile similar to secondary prevention	Presence signals higher risk
Other biomarkers	Cardiac troponins, natriuretic peptides, inflammatory markers	May enhance risk classification; routine use in primary care is limited
Type 2 diabetes mellitus	Increases risk due to multiple pathophysiological mechanisms	May be considered in adults with diabetes at increased CVD risk, after shared decision-making on bleeding risk vs. mild benefit
Chronic kidney disease	Later stages are associated with higher CVD risk; primary prevention evidence is less direct and must be weighed against increased bleeding risk	Benefit may be population-dependent
Metabolic dysfunction-associated steatotic liver disease	Associated with increased CVD risk; evidence relates mainly to liver fat and hepatocellular carcinoma outcomes, not direct CVD prevention.	Evidence is emerging; generalizability is limited
Familial hypercholesterolemia	Markedly increased LDL from birth; high risk for ASCVD	Aspirin may be considered if other risk factors are present

ASCVD, atherosclerotic cardiovascular disease; CAC, coronary artery calcium; CPS, carotid plaque score; CT, computed tomography; CVD, cardiovascular disease; LDL, low-density lipoprotein; Lp(a), lipoprotein(a).

**Table 3:** Scenarios of high bleeding risk with aspirin use.

Scenario/Risk Factor	Description/Example Patients	Notes/Mitigation Strategies
Advanced age (≥75 years)	Older adults	Consider PPI co-therapy; for upper gastrointestinal bleeding risk; assess overall bleeding risk, including intracranial hemorrhage, which may outweigh benefit
Asian or Pacific ancestry	Patients of Asian or Pacific descent	Increased bleeding risk vs. European ancestry
History of prior bleeding	Previous GI bleed, intracranial hemorrhage, etc.	Avoid aspirin or use with extreme caution
Peptic ulcer disease	Active or previous ulcers	PPI co-therapy and *H. pylori* eradication may reduce upper gastrointestinal bleeding risk
Chronic liver disease or pancreatitis	Cirrhosis, hepatitis, alcohol-related liver disease	High risk; avoid unless compelling indication
Cancer history	Especially GI or hematologic cancers	Individualized assessment required
Current smoking	Active smokers	Smoking cessation advised
Diabetes mellitus	Especially with other risk factors	Assess bleeding vs. cardiovascular risk
Alcohol-related disorders	Heavy drinkers, alcohol abuse	Avoid aspirin if possible
Concomitant medications	NSAIDs, corticosteroids, SSRIs, anticoagulants	Minimize use of interacting drugs; consider PPI co-therapy where upper gastrointestinal bleeding risk is elevated
*H. pylori* infection	Documented infection	Eradication therapy may reduce upper gastrointestinal bleeding risk

GI, gastrointestinal; *H. pylori*, *Helicobacter pylori*; NSAID, nonsteroidal anti-inflammatory drug; PPI, proton pump inhibitor; SSRI, selective serotonin reuptake inhibitor.

## Data Availability

No new data were created or analyzed in this study. Data sharing is not applicable to this article.
